# Evolutionary origins and specialisation of membrane transport

**DOI:** 10.1016/j.ceb.2018.06.001

**Published:** 2018-08

**Authors:** Joel B Dacks, Mark C Field

**Affiliations:** 1 Department of Cell Biology, Faculty of Medicine and Dentistry, University of Alberta, Edmonton, Alberta T6G 2H7, Canada; 2 School of Life Sciences, University of Dundee, Dundee DD1 5EH, UK

## Abstract

From unicellular protists to the largest megafauna and flora, all eukaryotes depend upon the organelles and processes of the intracellular membrane trafficking system. Well-defined machinery selectively packages and delivers material between endomembrane organelles and imports and exports material from the cell surface. This process underlies intracellular compartmentalization and facilitates myriad processes that define eukaryotic biology. Membrane trafficking is a landmark in the origins of the eukaryotic cell and recent work has begun to unravel how the revolution in cellular structure occurred.

**Current Opinion in Cell Biology** 2018, **53**:70–76

This review comes from a themed issue on **Membrane trafficking**

Edited by **Anne Spang** and **Satyajit Mayor**

For a complete overview see the Issue and the Editorial

Available online 19 June 2018

**https://doi.org/10.1016/j.ceb.2018.06.001**

0955-0674/© 2018 The Authors. Published by Elsevier Ltd. This is an open access article under the CC BY license (http://creativecommons.org/licenses/by/4.0/).

## The sophisticated last eukaryotic common ancestor

Many studies of membrane trafficking evolution focused on determining the organelles and proteins present in the last eukaryotic common ancestor (LECA), a hypothetical organism living ∼10^9^ years ago. As discussed in detail elsewhere [1,2], the numbers of predicted transport pathways and/or components within the LECA likely exceeded many extant organisms and LECA possessed all the canonical endomembrane organelles [1], extending to a *cis*, medial and *trans*-cisternal differentiated Golgi complex [3^•^]. These inferences also provide insights into the basic mechanisms of vesicle formation and fusion [1].

LECA is deduced to have possessed at least nine distinct vesicle coat complexes (including clathrin/AP1-5, COPI, TSET, COPII, retromer, ESCRT), ARF/ARF-like GTPases and their regulators [1,2]. LECA also possessed complex fusion machinery, including an extensive SNARE complement [4^••^], multisubunit tethering complexes [5], Rab GTPases [6, 7, 8] and regulatory factors [9]. Thus, LECA was capable of endocytosis, secretion and complex sorting, and while this is perhaps surprising, metabolism, cytoskeleton, mitochondrial functions, nuclear transport and many other cellular systems demonstrate similar predicted complexity.

As new components and pathways are discovered within transport and sorting machinery, their relevance to LECA and subsequent evolution can be addressed, for example recent descriptions of vesicle formation machinery such as TSET [10], Tepsin [11,12^•^], TSSC1 [13^•^] and novel clathrin adaptors [14^•^]. As a complex LECA should now be taken as a starting assumption, within trafficking systems and elsewhere, this complexity leads to two questions; what preceded LECA and how has subsequent evolution unfolded?

## How did complex membrane trafficking evolve?

**Figure 1:**
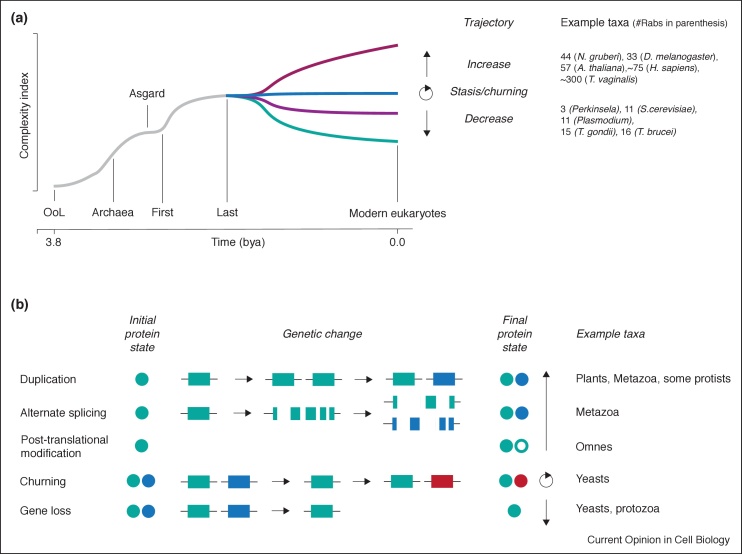
Generation of complexity. **(Panel a)** Timeline for alterations to complexity with cells and genomes across the history of life, with emphasis on the eukaryotic lineage. The `Complexity Index’ is an abstract concept to express compartmental sophistication, and is assumed to have increased during the ascent of eukaryotes (gray line), including the contributions from the archaea and bacterial (not shown) donors. Significantly, there are examples of extant taxa where the number of compartments, as predicted by the size of the Rab gene cohort, have increased (red), decreased (purple, teal) or remained approximately constant (blue) from the predicted state in the LECA. OoL; Origin of life. Note that points of complexity increase, as well as the curve topology, are arbitrary and for illustrative purposes; the true topology is unknown. **(Panel b)** Mechanisms for the generation of molecular complexity, based on examples from Rab protein evolution. Proteins are shown as circles, and genes as rectangles. The open circle denotes a modified protein, for example phosphorylated or acetylated.

The most basic evolutionary question, how did an endomembrane system originate, cannot be resolved by reconstructing LECA, as this represents an already advanced cell. As we assume complexity arose from a simpler state, this implies that transition from the first eukaryotic common ancestor (FECA; the first differentiated lineage from archaea giving rise only to organisms possessing some eukaryotic traits) to LECA required a revolution in cellular mechanisms (Figure 1). Promisingly, details of this revolution are now being discerned [15].

The basic vesicle trafficking machinery involves several protein families, each member of which functions at a specific organelle or transport pathway. Furthermore, organelle and pathway identity arises via combinatorial protein–protein interactions [16]. Different combinations of Rabs, SNAREs and tether complexes interact and substituting one or a few components can alter intracellular localisation. As these families evolved via gene duplication (and subsequent neofunctionalisation (Figure 1)), a mechanism for organelle evolution can be proposed; that organelle complexity arose where a primordial set of vesicle formation and fusion proteins allowed for transport and, through gene duplications and co-evolution of interacting proteins, developed new specificity. One pathway became two, and by simple iteration, many. This mechanism, the `organelle paralogy hypothesis’, found experimental support and has been elaborated upon repeatedly since the original proposal [2,9,17^•^,^18^,19^•^,^20^,^21^].

An important corollary to the concept of trafficking complexity emerging via incremental steps based on gene duplications is that, if the order of these steps can be resolved, the order in which the pathways originated would emerge. While significant challenges remain, over two-thirds of predicted LECA Rabs fall into either an endocytic or broadly secretory grouping [7]. Adaptins can be resolved to allow inference of multiple independent origins of pathways for internalization from the cell surface and post-Golgi transport [10]. Similar information for any membrane trafficking protein can, theoretically, determine the order of organelle origins, presently an exciting prospect.

**Figure 2:**
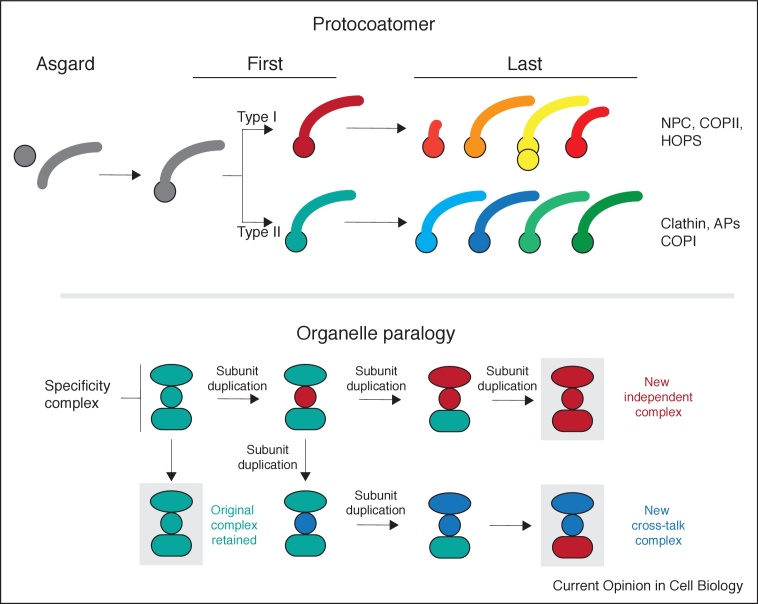
Paradigms for molecular evolution of eukaryotic cellular compartments and function. **(Top)** The protocoatomer hypothesis is predicated on the recognition that components of many membrane coating complexes share a particular architecture, specifically β-propeller plus α-solonoid secondary structural elements. While α and β structural elements are obviously common and present in all genomes, the combination of an N-terminal β-propeller plus α-solonoid configuration appears to be a hallmark of membrane deforming complexes, as well as being a eukaryotic signature. Complexes incorporating these proteins include the classical coats (COPI, COPII, clathrin), as well as the nuclear pore complex (NPC), adaptins and several others. Current evidence supports the following model: β + α proteins are encoded on the same cistron in Asgard archaea (but not as a single gene), which are presumed to have become fused at least by the time of the first eukaryotic common ancestor (First). Evidence suggests that there are at least two distinct types of protocoatomer in extant eukaryotes, based on the presence of several distinct accessory domains as well as structural criteria; these are arbitrarily termed type I and type II. It is unclear when these arose, but presumed an early event in transitioning from the first to last (Last) eukaryotic common ancestor (red/teal). Subsequent paralog expansions led to many distinct coats. Type I, which contains the NPC and COPII, appears more structurally diverse than Type II. Significant details for protocoatomer evolution remain to be determined. β-propeller structures are represented by a circle and α-solonoid by a curved bar of varying length. **(Lower)** Expanded version of the organelle paralogy hypothesis. Organelles are defined by the presence of members of paralog families, which include the Rab and SNARE proteins. Here we assume that three proteins (teal) are sufficient to define an organelle. Duplication of the circular factor allows the second copy (red) to neofunctionalise, as the original complex is retained. Initially this factor is able to interact with all of the original complex factors, but mutations will facilitate a change in specificity and the ability to bind a red eclipse and subsequently a red rounded rectangle. These latter factors are also the products of paralog expansion. Further mutation of the red circle (blue) can allow both a similar trajectory as before, as well as the possible sharing of components, again assisted by the paralogous nature of the various components.

While adaptin complex genes are easily identifiable, a deep connection is also likely present between many additional coat proteins. Homology here is based on retention of one or more β-propellers followed by an α-solenoid, a ‘protocoatomer’ configuration, and members can be grouped into two subfamilies, with distinct structural features (Figure 2). Importantly this encompasses more complexes than classic coat proteins and includes the nuclear pore and SEA complexes and intraflagellar transport system, uniting the origins of most nonendosymbiotic organelles [22^••^]. Reconstructing the evolutionary relationships remains a tremendous challenge due to massive sequence divergence and recent attempts have provided only partial solutions (e.g. [23^•^]). Further, the NPC may contain multiple protocoatomer subfamily architectures, suggesting an origin post-dating establishment of several organelles [24,25,26^•^].

There are relatively few hypotheses for membrane trafficking’s ultimate origin, and most are part of models explaining the origin of the eukaryotic cell itself [27, 28, 29, 30, 31,32^••^]. The best of these suggest both a coherent model as well as incorporate existing data objectively. As new data arises, for example the demonstration that hybrid archael and bacterial lipid membranes are biochemically and biologically viable [33^••^], some theories need to be modified or discarded in favour of hypotheses better supported by data. Perhaps the most available data at present, and thus best incorporated into models is the phylogenetic affinity and the relevant origins of membrane-trafficking components. For the overwhelming majority of these proteins, origins are of archaeal ancestry, supporting an autogenous evolution rather than endosymbiosis.

This model was provided a major boost with descriptions of the Lokiarchaeota [34] and subsequently a larger clade of related taxa, the Asgardarchaeota [35^••^]. Asgardarchaeota biology is inferred via metagenomic assemblies from locales as exotic as deep-sea sediments and thermal springs to those as mundane as marine estuaries. Very recent environmental surveys have uncovered additional candidate Asgardarchaea-related sequences suggesting wide prevalence [36]. Phylogenetics indicates that eukaryotes emerged from within the Asgardarchaea, that is, that many Asgardarchaea genes are the most similar of all prokaryote sequences to their eukaryote descendants. These metagenomes encode proteins previously considered eukaryote-specific, including ESCRT subcomplex I and II components, longin domain-containing proteins, expanded GTPase families similar to Rabs and Arf-like superfamilies, [37,38,39^•^], putative COPII components and possible protocoatomer-related proteins. These features are consistent with the Asgardarchaea as an ancestral source for many membrane-trafficking components [35^••^,^37^,^38^]. However, technical and methodological concerns regarding these genomes have been raised which need to be addressed, not least the authenticity of the metagenomic assemblies as derived from a single species (see [40^••^,^41^,^42^] for the latest in this debate). Isolation and culturing of an Asgardian remains crucial for evaluating their contribution to eukaryogenesis.

## How has the complex membrane trafficking system modified in LECA’s descendants?

Understanding the origins of LECA complexity is one part of evolutionary study of membrane-trafficking; the counterpart is defining processes that shaped complexity post-LECA and what diversity has since arisen. Some components, for example, COPI and AP1 complexes, are near ubiquitous [10], suggesting they are both ancient and indispensable. Other components expanded in certain lineages or introduced novel domains [14^•^,43^•^]. Other components still, such as AP5 [44] and DSCR3 [45], are present in organisms spanning eukaryotic diversity, but frequent losses suggest that these ancient complexes are expendable, under some conditions. Several components (e.g. the SNARE NPSN) are lost from animals and fungi, indicating that opisthokonts have lineage-specific gains and losses, just as any other. While parasite genomes tend to be reduced, there are striking examples of gene family expansions in *Entamoeba* [46, 47, 48] and *Trichomonas* [48,49].

Inferring biology from genome sequence implies functional homology, that is, that a given gene retains the same function in different lineages. Evidence supports this for many gene products, including Rab5, 7 and 11, AP1 and 2 and ESCRT (reviewed extensively in [37,38]). Examples where functional homology is less apparent include organelles absent from animals or fungi, such as the osmoregulatory contractile vacuoles and modified secretory lysosomes associated with predation or parasitism, for example, mucocysts in ciliates and rhoptries in apicomplexa.

## Trypanosomatids: a detailed case study

**Figure 3:**
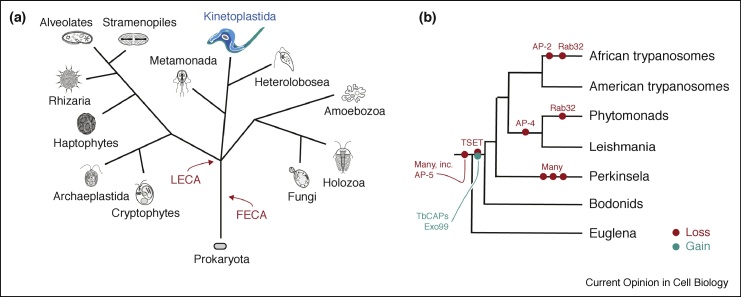
Phylogeny of eukaryotes, emphasising the kinetoplastida. **(Panel a)** The tree is based on most recent views of eukaryotic relationships, and each clade is accompanied by an illustrative diagram for a representative species. Positions of LECA and FECA are indicated in red, and kinetoplastida are highlighted in teal. **(Panel b)** Evolutionary relationships between the organisms discussed in the text, together with indications of where reconstruction suggest various proteins have been lost or gained during evolution.

Understanding the extent to which adaptations are associated with smaller scale changes requires fine-scale investigations coupling genomics and cell biology. One well studied group of protists are the Euglenozoa, which are distantly related to animals and fungi (Figure 3). A vast array of lifestyles within the lineage, ranging from the free living and photosynthetic *Euglena* spp., [57] to *Bodo saltans*, [58^•^] a phagophore, and many parasitic forms, including trypanosomes and *Leishmania*, provides a perfect opportunity for evaluating predictions of post-LECA trafficking trends. High quality genome and experimental resources allow this to be investigated with some rigour.

Trypanosome endocytosis is exclusively clathrin-mediated, with an intriguing amalgam of conserved proteins, for example, epsinR, CALM, AAK and AP-1, losses, for example, Dab2, and lineage-specific innovations conserved throughout kinetoplastids, for example, TbCAPs [14^•^,^50^,^51^]. An emerging paradigm is of a conserved core with a secondary ‘shell’ of lineage-specific proteins, *albeit* frequently retaining common architectural features. For example TbCAP80 and TbCAP141 are both phosphoinositide-binding proteins with an N-terminal lipid-interacting domain and disordered C-terminus, similar to organisation of ANTH and ENTH proteins [14^•^,^51^]. Similarly, kinetoplastid exocytic pathways are modified, with an additional lineage-specific subunit, Exo99, as a component of an otherwise conventional octameric exocyst Boehm *et al.* [52^•^] The functional implications of these innovations remain cryptic.

A major overall trend across the lineage is of secondary loss in the Rab and SNARE proteins, *albeit* following a gradual shift in complexity. Kinetoplastids retain essentially all SNARE proteins predicted to be present in LECA, but lack several Rabs, including Rab8, 34 and 50. Whilst the former functions in post-Golgi transport, Rab34 and Rab50 are uncharacterised. Simplification of anterograde pathways is unsurprising as exocytosis in trypanosomes does not appear to be significantly differentiated into multiple pathways. Within the kinetoplastids, *Bodo saltans* has the largest Rab and SNARE gene complement, which undergoes a gradual diminishment as one progresses through the Leishmanias, American trypanosomes (*Trypanosoma cruzi* and relatives) and finally to African trypanosomes (*T. brucei* and relatives) (Figure 3). Significantly, the plant parasitic Phytomonads are also reduced. These alterations of trafficking complexity likely reflect life style; for example *B. saltans* must adapt to rapid environmental and nutrient changes and has a large repertoire of Rab7 and Rab32-related Rabs facilitating autophagic and complex digestive functions, as well potentially as the osmoregulatory contractile vacuole. *Leishmania* and *T. cruzi* invade host cells and retention of a more complex transport system by *T. cruzi*, may reflect this and specifically a need to adapt and exploit autophagic mechanisms if resources are scarce. Both Phytomonads and African trypanosomes are distinguished by remaining extracellular in their respective plant and mammalian hosts; it is probably significant that both lack Rab32, have very few lineage-specific Rab proteins and also, in the case of Phytomonas, have a significant loss of the endocytic Rab21 and 28. Significantly, African trypanosomes lost the AP-2 complex as an adaptation to antigenic variation and a need for extremely rapid endocytosis for immune evasion [53]. However, recently we found that *T. cruzi*, which does possess the genes for all AP-2 subunits, apparently does not use this complex for endocytosis [54], indicating that AP-2 independent endocytosis is more widespread than inferred by comparative genomics and perhaps serves to underscore the importance of experimental study. Most recently, the genome sequence for *Perkinsela*, an intracellular kinetoplastid parasite with a greatly reduced genome has been reported; this organism has but three Rab1/Rab2-like proteins [55^••^], providing a provocative example of the flexibility of the trafficking system and its evolution.

## Conclusion

Eukaryotic diversity is immense and has direct bearing upon our health, agriculture and environment. Understanding how such distinctiveness came to be is a major goal of evolutionary cell biology [56]. Genome sequencing, direct experimentation and increased sampling of environments have all revealed pathways that shaped the multiplicity of cellular forms and architectures, with membrane trafficking retaining a position centre stage. With considerable knowledge, we are in the exciting position of beginning to understand the origins of trafficking, and to explore the many facets of these pathways in multiple lineages.

## References and recommended reading

Papers of particular interest, published within the period of review, have been highlighted as

• of special interest
•• of outstanding interest
